# Effect of nocturnal hemodialysis on sleep parameters in patients with end-stage renal disease: a systematic review and meta-analysis

**DOI:** 10.1371/journal.pone.0203710

**Published:** 2018-09-11

**Authors:** Lingzhi Li, Xi Tang, Sehee Kim, Ye Zhang, Yi Li, Ping Fu

**Affiliations:** 1 Division of Nephrology, Kidney Research Institute, West China Hospital of Sichuan University, Chengdu, China; 2 Department of Biostatistics, School of Public Health, University of Michigan, Ann Arbor, MI, United States of America; 3 Sleep Medicine Center, West China Hospital of Sichuan University, Chengdu, China; 4 Kidney Epidemiology and Cost Center, School of Public Health, University of Michigan, Ann Arbor, MI, United States of America; 5 West China Biostatistics and Cost-Benefit Analysis Center, West China Hospital of Sichuan University, Chengdu, China; Medical University of Gdansk, POLAND

## Abstract

**Introduction:**

Recently, a small but growing literature has depicted the beneficial effects of nocturnal hemodialysis (NHD) over conventional hemodialysis (CHD) in the fields of sleep disorders such as sleep apnea. The impact of various dialysis models on sleep disorders, however, has not been determined. The objective of our meta-analysis is to examine the potential effects of NHD, compared with CHD, on sleep disorders in HD patients.

**Methods:**

Several electronic databases including PubMed, EMBASE, Cochrane Central Register of Controlled Trials, ClinicalTrials.gov and CNKI were searched, using the search terms “nocturnal” (or “nightly”) and “dialysis” (or “hemodialysis” or “renal dialysis”) from the earliest available date of indexing to March 2018. Two authors independently extracted data, evaluated the study quality, and conducted random-effects meta-analyses using STATA 12.0.

**Results:**

Of 1789 potentially relevant citations, 9 fulfilled eligibility criteria, consisting of 6 single-arm studies (comparing pre- and post-intervention outcomes), 1 observational study, and 2 randomized controlled trials (a total of 286 participants). Regarding objective sleep assessments, conversion from CHD to NHD resulted in a significant reduction in the AHI (Mean difference was -14.90; 95% CI, -20.12 to -9.68), a significant increase of SaO2 (Mean difference was 1.38%; 95% CI, 0.35% to 2.42%), and a significant decrease of TST (Mean difference was -0.31; 95% CI, -0.47 to -0.15). The trends were even stronger in the HD patients with sleep disorders. However, regarding subjective sleep assessments, improved sleep quality was found in the prospective pre-post intervention studies and cohort studies, while no significant improvements were found in the randomized controlled trials.

**Conclusion:**

Although a significant improvement of sleep apnea was observed by switching from CHD to NHD, it may not yield a net benefit in overall subjective sleep quality.

## Introduction

Sleep-disordered breathing (SDB) is highly prevalent and increasingly recognized among patients with end-stage renal disease (ESRD) [1]. Sleep apnea, one kind of SDB, is a risk factor for cardiovascular diseases and may contribute to the mortality and morbidity in the ESRD population [2, 3]. The prevalence of sleep apnea in ESRD patients is higher than 50% [4], which is at least ten times higher than the prevalence reported in the general population [5]. However, the pathogenesis of sleep apnea in ESRD remains unclear. Previous work on features of sleep apnea in ESRD patients [6–9] suggested that its pathogenesis may be related to both upper airway occlusion and destabilization of central respiratory control.

For ESRD patients, the most common therapy is conventional hemodialysis (CHD), also known as intermittent hemodialysis, which conducts dialysis 3 days per week with each session lasting 4–5 hours. Nocturnal hemodialysis (NHD) is a novel and intensive form of renal replacement therapy, which occurs 4–7 nights per week with each session lasting 5–8 hours at home or in a center [10]. In the past ten years, a small but growing number of studies have shown NHD is superior to CHD in reversing many of the physiologic perturbations of uremia [11–15]. It increases small solute clearance like phosphate and improves hemoglobin, sleep patterns, nutrition and quality of life, which in turn reduces cardiovascular risks and hospitalization rates [16, 17]. A recent meta-analysis based on 21 studies including a total of 1,165 in-center nocturnal HD patients and 15,865 conventional HD patients showed improvements in systolic blood pressure, increase of hemoglobin levels, and decrease of serum phosphate levels [18]. These findings are consistent with another meta-analysis based on 46 studies, reporting that the switch from CHD to frequent or extended HD (including NHD) improves cardiovascular parameters and may provide long-term cardiovascular benefits [19]. Other meta-analyses showed that, compared with CHD, NHD is associated with a higher protein and energy intake, higher serum albumin, better survival, and better quality of life [20–22].

During the last decades, several studies have been conducted to directly compare NHD with CHD on sleep parameters [6, 8, 9, 23, 24]. Recently, another meta-analysis specifically reviewed studies about the association between renal replacement therapy (RRT) modality and sleep apnea and found intensive RRT (transplant, continuous cycler-assisted peritoneal dialysis or intensive HD) has a lower risk of sleep apnea than the standard/conventional RRT (CHD or continuous ambulatory peritoneal dialysis) [25]. However, the existing systematic reviews or meta-analyses did not study the effect of NHD on sleep disorders from both subjective and objective aspects. Moreover, the results have been inconsistent, and therefore inconclusive, in part due to differences in study populations and small sample sizes.

This systematic review and meta-analysis is aimed to examine the potential effects of NHD, compared to CHD, on sleep disorders in HD patients. We examined sleep-quality-related scores, both subjective and objective.

## Materials and methods

### Data sources and searches

Publications were identified by searching electronic databases including PubMed, EMBASE, Cochrane Central Register of Controlled Trials, ClinicalTrials.gov and CNKI from the earliest available date of indexing to March 2018. The search terms to identify eligible studies included “nocturnal” (or “nightly”) and “dialysis” (or “hemodialysis” or “renal dialysis”). In addition, the references cited by the identified original studies and related review articles were searched manually as well. The search was limited to human studies.

### Study selection and sleep outcomes

All types of studies comparing the effect of NHD with CHD on sleep disorders were initially considered, except for case report. Studies of hemofiltration, hemodiafiltration and peritoneal dialysis were excluded. The outcomes of interest were objective or subjective changes in sleep parameters including apnea-hypopnea index (AHI), mean oxygen saturation during sleep (SaO2), total sleep time (TST), and scores by sleep questionnaire or interviews. The AHI is defined as the total number of apneas and hypopneas during sleep divided by the total number of sleep hours. There were no restrictions on language, sample size, or duration of follow-up. Two authors (Li and Tang) screened the titles and abstracts of all electronic references and retrieved the full-text articles for comprehensive review independently. Any disagreement in selecting studies was discussed with other authors and resolved by consensus.

### Data extraction and quality assessment

The two authors (Li and Tang) who reviewed all the study characteristics (first author, year of publication, country of origin, study design, sample size, percentage of men, age, duration of CHD dialysis, duration of study and methods to measure sleep related parameters) independently collected and extracted the data. The detailed information on NHD and CHD treatment such as frequency per week and duration of the session was extracted. To assess the severity of sleep apnea, sleep parameters, including the AHI, SaO2, and TST (measured by Polysomnography (PSG) or Actigraphy), were extracted.

For the quality assessment of randomized controlled trials (RCTs), Cochrane Collaboration’s tool was used [26]. For the quality assessment of non-randomized studies, Newcastle-Ottawa Scale was used, where this scale assigns a maximum of nine stars to a study based on the quality of patient selection, comparability, exposure, and outcome [27]. The quality of each study was independently evaluated by two reviewers (Li and Tang). Each reviewer scored the included studies as ‘high quality’ (if scored 7–9 stars) or ‘medium quality’ (if scored 4–6 stars). Any discrepancies were determined by consensus.

### Statistical analysis

We meta-analyzed the effect of switch from CHD to NHD on sleep parameters. Hence, mean differences (MDs) in sleep parameters between the two treatments were combined for the analysis [19, 28]. When the standard error of the change was not directly reported, we estimated it from the standard errors of the baseline (pre-NHD) and final values by assuming that the correlation between the baseline and final values was 0.5 [19]. The existence of heterogeneity among effect sizes was evaluated by the I^2^ index, which indicates medium to high heterogeneity if the I^2^ index is over 50% [28]. Two-tailed P-value <0.05 was considered statistically significant, and a 95% confidence interval (CI) was provided for a mean difference. The meta-analyses were performed using STATA 12.0 with metan commands. All analyses were conducted in parallel by two investigators (Li and Tang). Due to the paucity of enrolled studies, the potential for publication bias could not be assessed by funnel plots or Begg’s test and Egger’s test.

Given medium to high heterogeneity existing in some of the outcome variables, we used a random effect model. In addition, subgroup analyses were also performed to explore possible sources of heterogeneity and clinical significance related to the following two characteristics: 1) presence of sleep disorders and 2) sleep parameter measurements during on- versus off-dialysis days.

## Results

### Study characteristics

**Fig 1:**
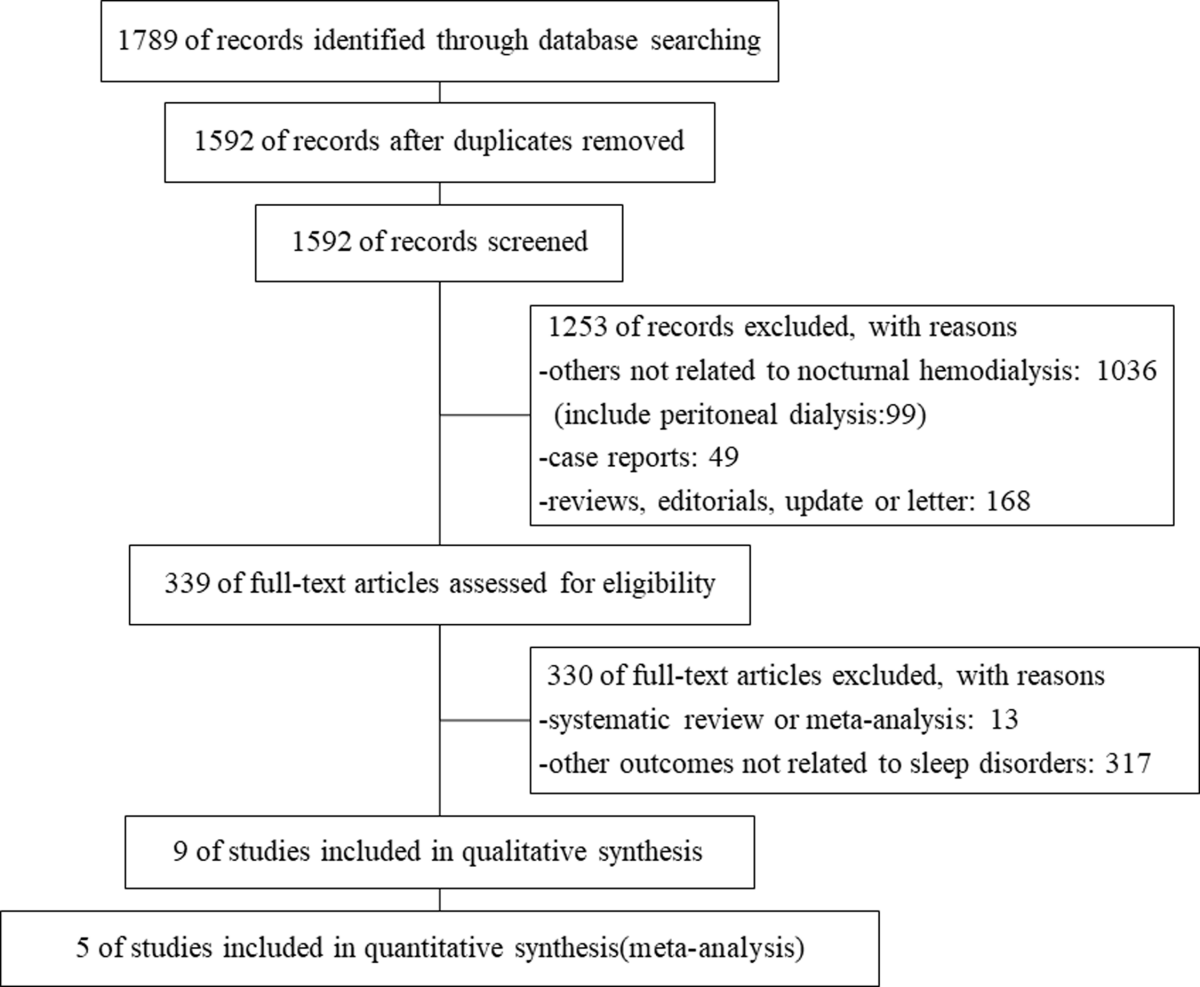
Flow chart for the study selection.

Fig 1 displays a flow chart outlining the search, review, and selection process for the studies included in this meta-analysis. A total of 1789 potentially related references were initially identified and screened, of which 339 articles were considered for full paper review. After careful selection, 10 studies fulfilled our inclusion criteria, of which 4 studies[6, 8, 9, 23] were from two study groups (Hanly et al and Beecroft et al). We decided to keep the two articles from Hanly et al [6, 23], because the sleep-related parameters in the two articles were totally different, which might implicate the enrolled patients were not the same. However, as for the two articles from Beecroft et al [8, 9] where the clinical characteristics (sample size, mean age, duration of CHD and duration of the study) of the two articles were almost the same, we only included the first one[8] in order to avoid the duplication of enrolled subjects. Finally, there were 9 studies included in our systematic review, which consisted of 6 single-arm studies, 1 observational study, and 2 randomized controlled trials (a total of 286 participants) [6, 8, 17, 23, 24, 29–32]. Characteristics of the individual studies are presented in Table 1. Quality assessment indicates that all studies were medium to high quality. The medium-quality was given to some studies due to lack of detailed description of follow-up.

**Table 1:** Characteristics of studies included in the systematic review and meta-analysis.

author (year)	country of origin	study design	sample size	percentage of men	mean age(years)	mean duration of CHD dialysis(months)	duration of study(months)	CHD	NHD	Objective/Subjective	study quality*	ref
**Hanly (2001)**	Toronto Canada	prospective pre/post	14	10(71.4%)	45±9	12 to 180	6 to 15	4 h/day, 3 days/week	at home 8–10 h/night 6–7 nights/week	PSG	6	[6]
**Hanly (2003)**	Toronto Canada	prospective pre/post	15	not mentioned	44±10	99.6±90	not mentioned	4 h/day, 3 days/week	at home 8–10 h/night 7 nights/week	PSG	6	[24]
**Chan (2004)**	Toronto Canada	prospective pre/post	9	5(55.6%)	44±2	not mentioned	6 to 15	4 h/day 3 days/week	at home 8–10 h/night 6 nights/week	PSG	6	[25]
**Culleton(2007)**	Alberta Canada	randomized controlled trial	52	32(61.5%)	CHD:53.1±13.4 NHD:55.1±12.4	not mentioned		3 days/week	at home 6 h/night 5–6 nights/week	HRQOL	6	[17]
**Beecroft (2008)**	Toronto Canada	prospective pre/post	24	15(62.5%)	overall:32 to 68 Apnoeic Responder:38±6 Apnoeic Non-responder:53±9 Non-apnoeic:40±6	overall:24.8 Apnoeic-Responder:19±17 Apnoeic-Non-responder:18±17 Non-apnoeic:38±73	3 to 6	4 h/day 3 days/week	at home 8 h/night 3–6 nights/week	PSG	6	[8]
**Koch (2009)**	The Netherlands	prospective pre/post	13	8(61.5%)	58[22]	25[20]	6	3–4 h/day 3 days/week	in center 8 h/night 4 nights/week	PSG sleep questionnaire	6	[30]
**Bugeja (2009)**	Ottawa Canada	prospective pre/post	39	26(66.7%)	49[39,63]	37.2[15.6,81.6]	22.8[4.8,33.6]	4 h/day 3 days/week	in hospital 7–8 h/night 3 nights/week	interview	7	[32]
**Koch (2010)**	The Netherlands	cohort study	CHD:20 NHD:13	CHD:14(70%) NHD:8(61.5%)	CHD:71[14.3] NHD:59[40]	CHD:19[20] NHD:25[20]	not mentioned	3-4h/day 3 days/week	in hospital 8 h/night 4 nights/week	Actigraphy sleep questionnaire	7	[31]
**Unruh (2016)**	USA Canada	randomized controlled trial	CHD:42 NHD:45	57(65.5%)	52.8±13.6	1 to 138	12	3 days/week	in hospital 6–8 h/night 3 nights/week	MOS SPI	7	[33]

Abbreviations: CHD, conventional hemodialysis; NHD, nocturnal hemodialysis. HRQOL, health-related quality of life questionnaires. Data are mean ± SD or median [interquartile range].

* Study quality of the cohorts or study arms was assessed by the Newcastle-Ottawa Scale. The quality of randomized controlled trial (RCT) was assessed by the Cochrane Collaboration’s tool.

In terms of the reported subjective sleep parameters, 5 studies assessed sleep quality based on different questionnaire or interviews [17, 29–32]. As the first objective sleep parameter, we considered AHI. Table 2 shows that 5 studies assessed the AHI [6, 8, 23, 24, 29], where either PSG or actigraphy was used to measure AHI. Three of these studies reported PSG results both on the day of dialysis and the off-dialysis day (the inter-dialysis period) [6, 23, 24]. In contrast, the remaining 2 studies reported PSG results of either on-dialysis day or off-dialysis day [8, 29], but not both. For patients with sleep disorders, there were only 3 studies [6, 8, 23] presenting AHI. As the second objective sleep parameter, we considered SaO2. Table 3 shows that 1 study assessed SaO2 both on- and off-dialysis days [6], while the other study assessed it only for off-dialysis day [8]. In both cases, a pulse oximeter was used. As the third objective sleep parameter, we considered TST. Table 4 shows that PSG or actigraphy was used in 5 studies to measure TST. TST was measured both on-dialysis day and off-dialysis day in 3 studies [6, 23, 24]. Only 2 studies reported results for the patients with sleep disorders [6, 23].

**Table 2:** Effects of nocturnal hemodialysis on apnea-hypopnea index (AHI).

First author (year)	Group		Dialysis*		AHI(n./h)
			CHD	NHD	
Hanly (2001) [6]	all patients		off	25±25	13±13
			on	25±25	8±8
	patients with sleep disorders		off	46±19	19±15
			on	44±22	9±9
Hanly (2003) [24]	patients with sleep disorders		off	18±26	10±7
			on	18±22	8±7
	patients without sleep disorders		off	29±22	15±16
			on	28±26	7±9
Chan (2004) [25]	all patients		off	29.2±9.9	7.2±3.3
			on	30.2±9.8	9.7±2.9
Beecroft (2008) [8]	patients with sleep disorders	Responder	off	42.5±23.9	7.3±4.2
		Non-responder	off	37.1±18.7	38.1±18.7
	patients without sleep disorders		off	7.5±3.8	16.0±19.6
Koch (2009) [30]	all patients		on	11.2[7.0]	5.6[6.8]

Abbreviations: AHI, apnea-hypopnea index; PSG, polysomnography; CHD, conventional hemodialysis; NHD, nocturnal hemodialysis. Data are mean ± SD or median [interquartile range].

* “On” indicates sleep parameters were measured during daytime for CHD and nighttime for NHD on on-dialysis day. “Off” indicates sleep parameters were measured at anytime during the inter-dialysis period for CHD and nighttime for NHD on off-dialysis day.

**Table 3:** Effects of nocturnal hemodialysis on mean oxygen saturation (SaO2).

First author (year)	Group		Dialysis*	Mean oxygen saturation (%)	
				CHD	NHD
Hanly (2001) [6]	all patients		off	93.8±2.0	94.7±1.9
			on	93.2±3.0	95.9±1.7
	patients with sleep disorders		off	92.6±2.0	93.7±1.6
			on	91.7±3.1	95.3±1.3
Beecroft (2008) [8]	patients with sleep disorders	Responder	off	92.3±1.3	96.9±2.3
		Non-responder	off	93.5±1.8	93.6±2.6
	patients without sleep disorders		off	94.4±2.1	95.1±1.9

Abbreviations: SaO2, oxygen saturation during sleep; PSG, polysomnography; CHD, conventional hemodialysis; NHD, nocturnal hemodialysis. Data are mean ± SD or median [interquartile range].

* “On” indicates sleep parameters were measured during daytime for CHD and nighttime for NHD on on-dialysis day. “Off” indicates sleep parameters were measured at anytime during the inter-dialysis period for CHD and nighttime for NHD on off-dialysis day.

**Table 4:** Effects of nocturnal hemodialysis on total sleep time (TST).

First author (Year)	Equipment	Group	Dialysis*	Total sleep time (TST)(h)	
				CHD	NHD
Hanly (2001) [6]	PSG	all patients	off	5.7±0.7	5.4±0.5
			on	5.6±0.7	5.0±0.6
		patients with sleep disorders	off	5.6±0.7	5.3±0.6
			on	5.8±0.5	5.2±0.7
Hanly (2003) [24]	PSG	patients with sleep disorders	off	5.7±0.6	5.3±0.6
			on	5.3±0.7	5.2±0.7
		patients without sleep disorders	off	5.5±0.8	5.4±0.7
			on	5.8±0.9	4.7±0.4
Chan (2004) [25]	PSG	all patients	off	5.4±0.2	5.3±0.2
			on	5.5±0.2	5.2±0.3
Koch (2009) [30]	PSG	all patients	on	6.95[0.82]	7.20[1.09]
Koch (2010) [31]	Actigraphy	all patients	on	6.13[0.32]	6.53[1.4]

Abbreviations: TST, total sleep time; PSG, polysomnography; CHD, conventional hemodialysis; NHD, nocturnal hemodialysis. Data are mean ± SD or median [interquartile range].

* “On” indicates sleep parameters were measured during daytime for CHD and nighttime for NHD on on-dialysis day. “Off” indicates sleep parameters were measured at anytime during the inter-dialysis period for CHD and nighttime for NHD on off-dialysis day.

### Effect of nocturnal HD on subjective sleep quality

For sleep quality, a meta-analysis was not performed since the questionnaires used to evaluate sleep quality varied from study to study, and some questions were based on subjective experiences. Instead, we will provide a systematic review summary of the existing studies. Three studies reported that overall subjective sleep quality on both dialysis and off-dialysis nights were improved after a switch from CHD to NHD [29–31]. Specifically, when compared with CHD, wake periods at night were shorter, and the intradialytic cramps and dizziness were ameliorated on NHD. In addition, patients on NHD were less exhausted during the daytime, and thus the quality of life has improved. However, other items in the sleep questionnaire, such as sleep-onset latency, estimated sleep time, and daytime naptime were not significantly different after the switch of dialysis modality. The two RCTs reported that there was no significant effect of NHD on self-reported sleep quality as well as self-reported hours of sleep and self-reported snoring or number of naps [17, 32].

### Effect of nocturnal HD on the sleep parameter AHI

**Fig 2:**
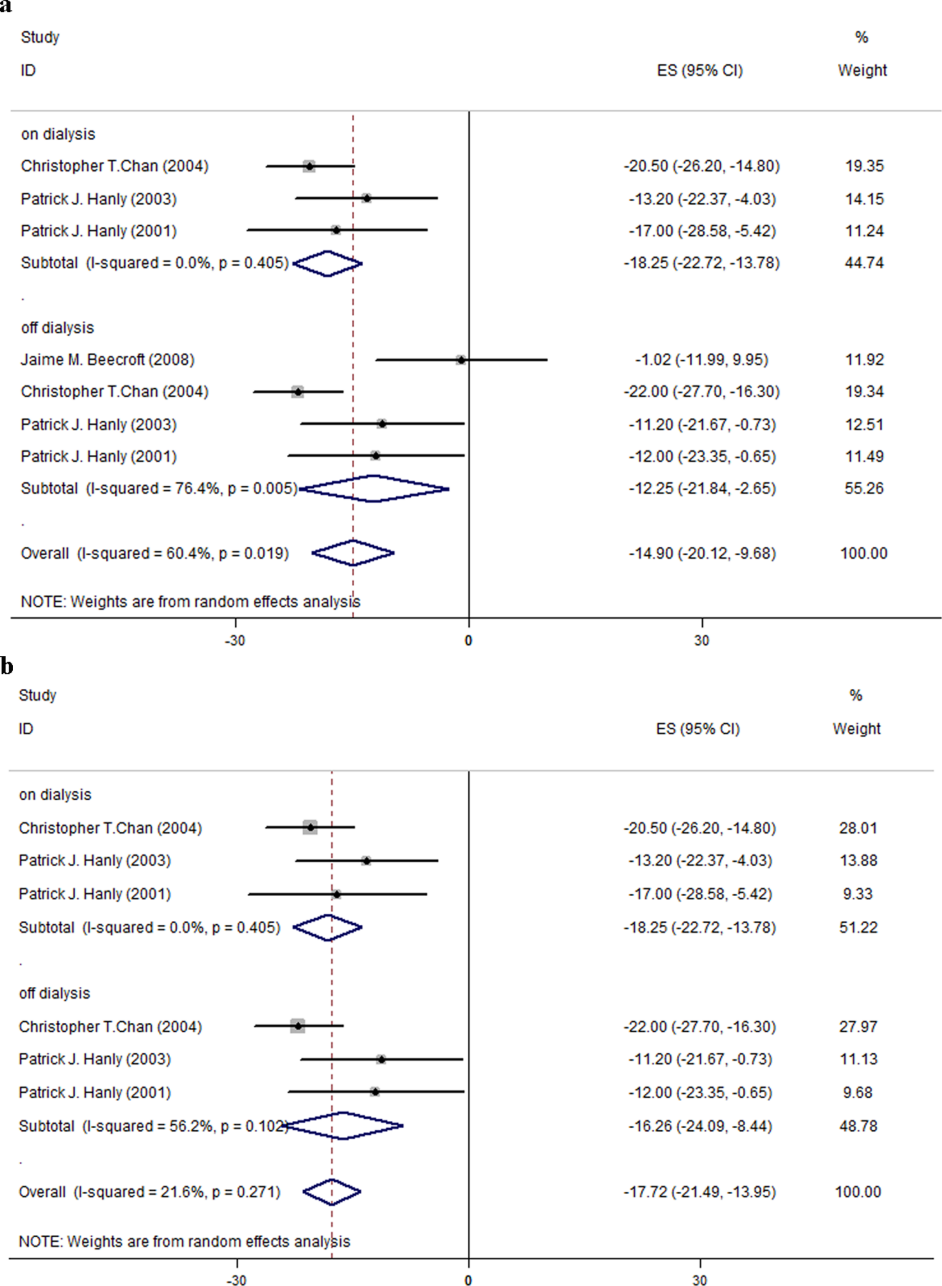
Changes of AHI in the studies for all patients. (a) Changes of AHI in the 4 included studies for all patients. (b) Changes of AHI in the 3 included studies for all patients: a sensitivity analysis.

In the meta-analysis of AHI, one study that used median and interquartile values to assess AHI was excluded [29]. The pooled results based on the remaining 4 studies (a total of 62 patients) [6, 8, 23, 24] are provided in Fig 2A. It shows that a switch from CHD to NHD yielded a significant reduction in the AHI (MD = -14.90; 95% CI, -20.12 to -9.68; I^2^ = 60.4%; Fig 2A) from the overall assessment combining on- and off-dialysis days in all HD patients. The magnitude of reduction in AHI during on-dialysis days and off-dialysis days, respectively, was -18.25 n./h (95% CI, -22.72 to -13.78; I^2^ = 0.0%) and -12.25 n./h (95% CI, -21.84 to -2.65; I^2^ = 76.4%). Fig 2B shows a sensitivity analysis result, where we further excluded the study by Beecroft et al. (2008) from the meta-analysis due to discrepancy in the dialysis frequency. The study allowed NHD occurring 3–6 days per week with each session lasting 8 hours (i.e., offered longer dialysis sessions than other studies). After the exclusion, the heterogeneity reduced from I^2^ = 60.4% to I^2^ = 21.6%, while the mean difference increased from MD = -14.90 to MD = -17.72 (Fig 2A and 2B).

**Fig 3:**
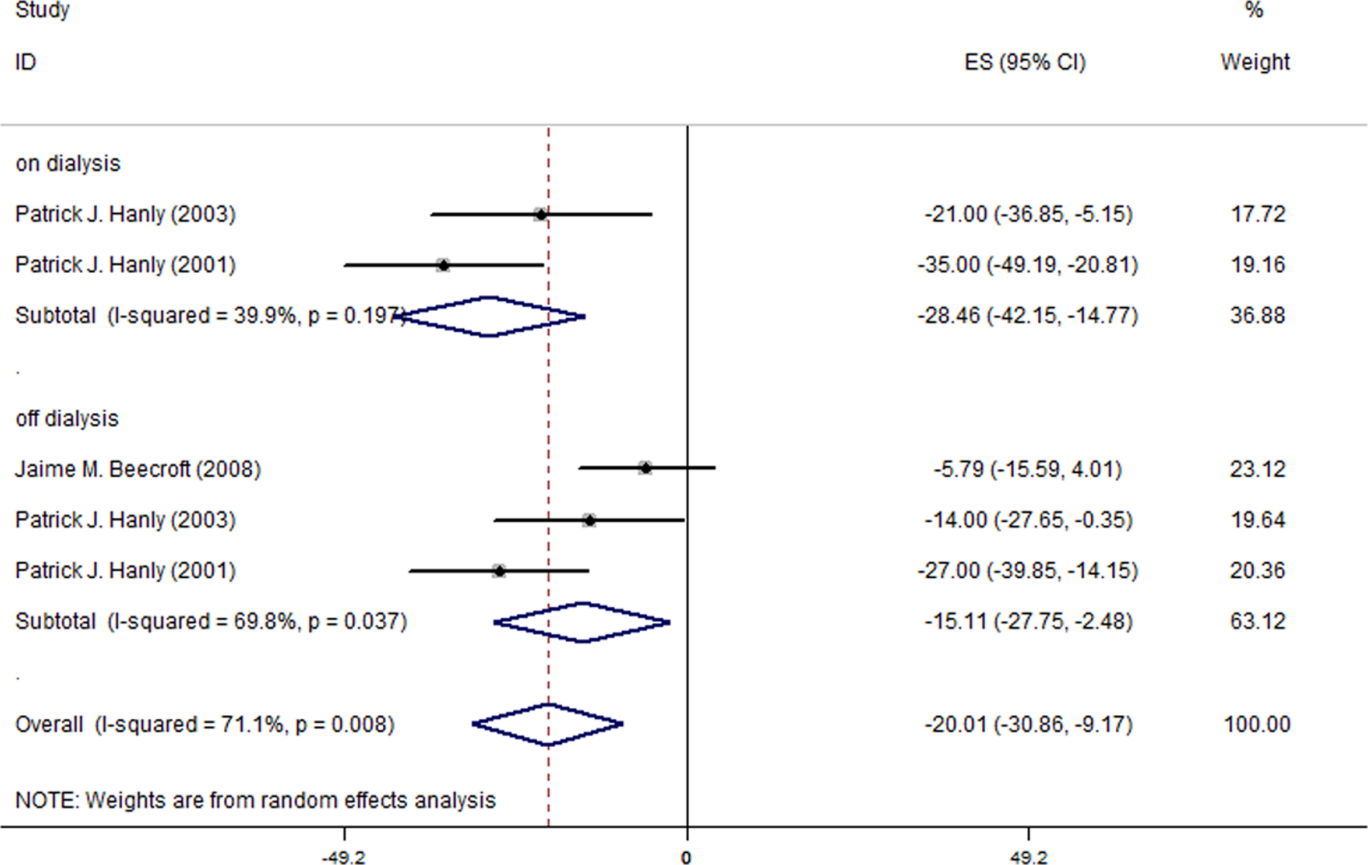
Changes of AHI in the 3 included studies for patients with sleep disorders.

Moreover, in the subgroup analyses for the patients with sleep disorders, AHI decreased to a greater degree (MD = -20.01; 95% CI, -30.86 to -9.17; I^2^ = 71.1%; Fig 3). The magnitude of reduction during on-dialysis and off-dialysis days was -28.46 (95% CI, -42.15 to -14.77; I^2^ = 39.9%) and -15.11 (95% CI, -27.75 to -2.48; I^2^ = 69.8%), respectively. In summary, AHI decreased more during on-dialysis days than off-dialysis days regardless of the presence of sleep-disorders (Table 5).

**Table 5:** Summary effects of nocturnl hemodialysis on AHI, SaO2, and TST.

Outcome variables	Group ^a^		No. studies	No. patients	Baseline mean value (95% CI)	Mean change ^b^ (95% CI)	P value	Assessment of heterogeneity	
								I^2^ index ^c^	P value
Apnea-hypopnea index (n./h)	all patients	overall	4	62	25.98 (22.90 to 29.06)	-14.90 (-20.12 to -9.68)	<0.001	60.4%	0.019
		on	3	38	25.29 (13.44 to 37.14)	-18.25 (-22.72 to -13.78)	<0.001	0.0%	0.405
		off	4	62	26.50 (22.55 to 30.44)	-12.25 (-21.84 to -2.65)	0.006	76.4%	0.005
	patients with sleep disorderss	overall	3	31	37.02 (26.70 to 47.34)	-20.01 (-30.86 to -9.17)	<0.001	71.1%	0.008
		on	2	15	36.00 (-65.65 to 137.65)	-28.46 (-42.15 to -14.77)	<0.001	39.9%	0.197
		off	3	31	37.70 (16.57 to 58.84)	-15.11 (-27.75 to -2.48)	<0.001	69.8%	0.037
Oxyhemoglobin saturation (%)	all patients	overall	2	38	93.55 (92.77 to 94.33)	1.38 (0.35 to 2.42)	<0.001	63.5%	0.065
		on	1	14	93.2	—	<0.001	—	—
		off	2	38	93.73 (92.77 to 94.68)	0.88 (0.20 to 1.56)	<0.001	0.0%	0.957
	patients with sleep disorders	overall	2	23	92.53 (90.56 to 94.49)	1.68 (0.28 to 3.09)	0.006	62.9%	0.068
		on	1	7	91.7	—	<0.001	—	—
		off	2	23	92.94 (88.65 to 97.23)	1.01 (0.11 to 1.91)	0.025	0.0%	0.866
Total sleep time (h)	all patients	overall	3	38	5.54 (5.42 to 5.67)	-0.31 (-0.47 to -0.15)	<0.001	59.2%	0.031
		on	3	38	5.52 (5.32 to 5.71)	-0.44 (-0.65 to -0.23)	<0.001	41.3%	0.182
		off	3	38	5.51 (4.17 to 6.84)	-0.14 (-0.26 to -0.03)	0.014	0.0%	0.410
	patients with sleep disorders	overall	2	15	5.60 (4.94 to 6.26)	-0.36 (-0.59 to -0.13)	0.002	0.0%	0.523
		on	2	15	5.55 (2.37 to 8.73)	-0.36 (-0.85 to 0.13)	0.154	53.5%	0.142
		off	2	15	5.65 (5.01 to 6.29)	-0.36 (-0.67 to -0.04)	0.027	0.0%	0.760

Abbreviations: AHI, apnea-hypopnea index; SaO2, Oxyhemoglobin saturation during sleep; TST, total sleep time.

^a^ “On” indicates sleep parameters were measured during daytime for CHD and nighttime for NHD on on-dialysis day. “Off” indicates sleep parameters were measured at anytime during the inter-dialysis period for CHD and nighttime for NHD on off-dialysis day.

^b^ By random effects model meta-analysis

^c^ A measure of statistical heterogeneity across study results an I^2^ index≥50% indicates medium-to-high heterogeneity.

### Effect of nocturnal HD on SaO2

**Fig 4:**
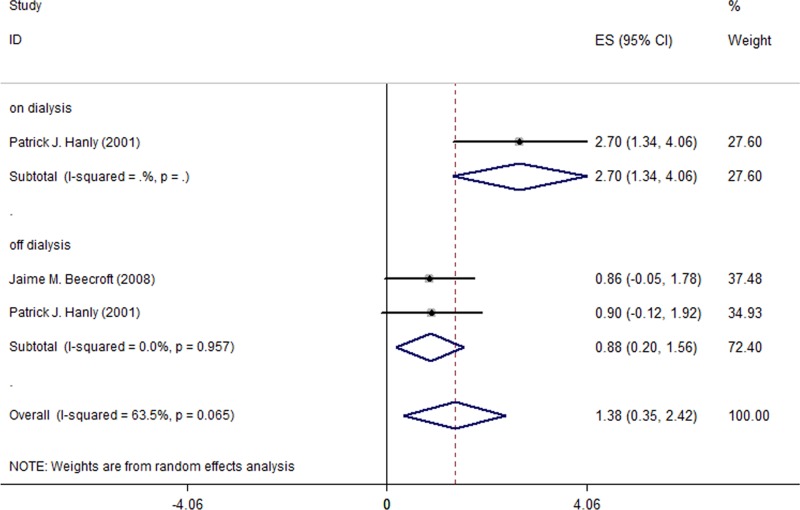
Changes of SaO2 in the 2 included studies for all patients.

Regarding SaO2, the meta-analysis pooling two studies (a total of 38 patients) showed a statistically significant increase after switch from CHD to NHD (MD = 1.38%; 95% CI, 0.35% to 2.42%; I^2^ = 63.5%; Fig 4). Furthermore, Table 5 shows that switching to NHD improved from the level below nominal, 95%, at baseline. During off-dialysis days, the meta-analysis showed that SaO2 increased by 0.88% (95% CI, 0.20% to 1.56%; I^2^ = 0.0%; Fig 4). With regard to on-dialysis days, however, only one study [6] reported SaO2 changes (a significant 2.7% increase; 95% CI, 1.34% to 4.06%) in the HD patients, and therefore a meta-analysis was not carried out.

**Fig 5:**
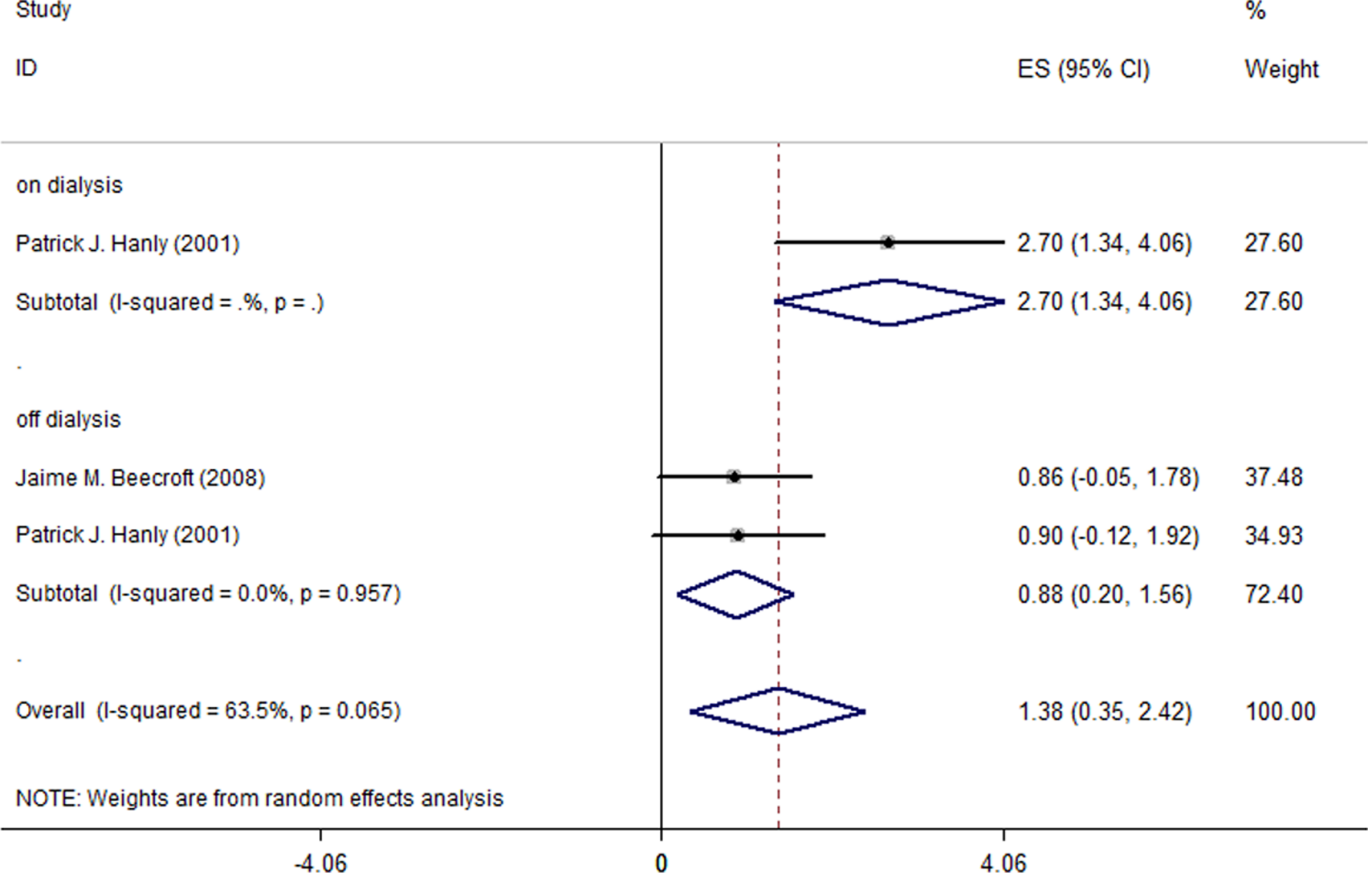
Changes of SaO2 in the 2 included studies for patients with sleep disorders using the combined data.

In the HD patients with sleep disorders, a larger improvement on SaO2 was observed after a switch from CHD to NHD. Fig 5 and Table 5 show that the increase in the off-dialysis SaO2 level was 1.01% (95% CI, 0.11% to 1.91%; I^2^ = 0.0%), and the increase in the combined on- and off-dialysis SaO2 level was 1.68% (95% CI, 0.28% to 3.09%; I^2^ = 62.9%).

### Effect of nocturnal HD on the sleep parameter TST

**Fig 6:**
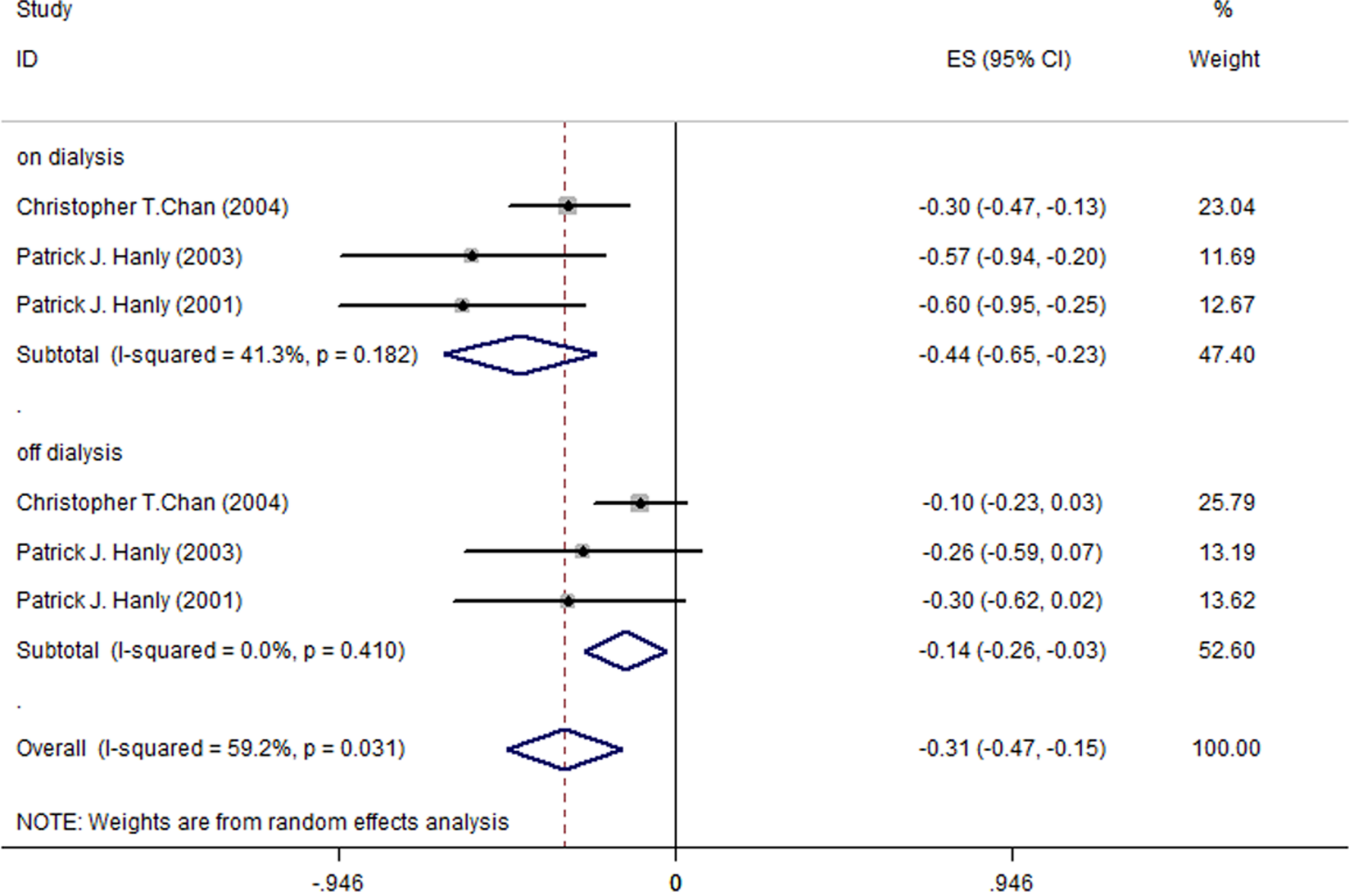
Changes of TST in the 3 included studies for all patients.

This meta-analysis included only 3 studies (a total of 38 patients)[6, 23, 24] among the 5 studied reporting results for TST outcome since the remaining 2 studies used a median and interquartile range to assess the NHD effect [29, 30]. Fig 6 shows that, after a switch from CHD to NHD, the TST change among all HD patients was -0.31h (95% CI, -0.47 to -0.15; I^2^ = 59.2%). TST decreased by -0.44h during on-dialysis days (95% CI, -0.65 to -0.23; I^2^ = 41.3%; Fig 6) and -0.14h during off-dialysis days (95% CI, -0.26 to -0.03; I^2^ = 0.0%; Fig 6).

**Fig 7:**
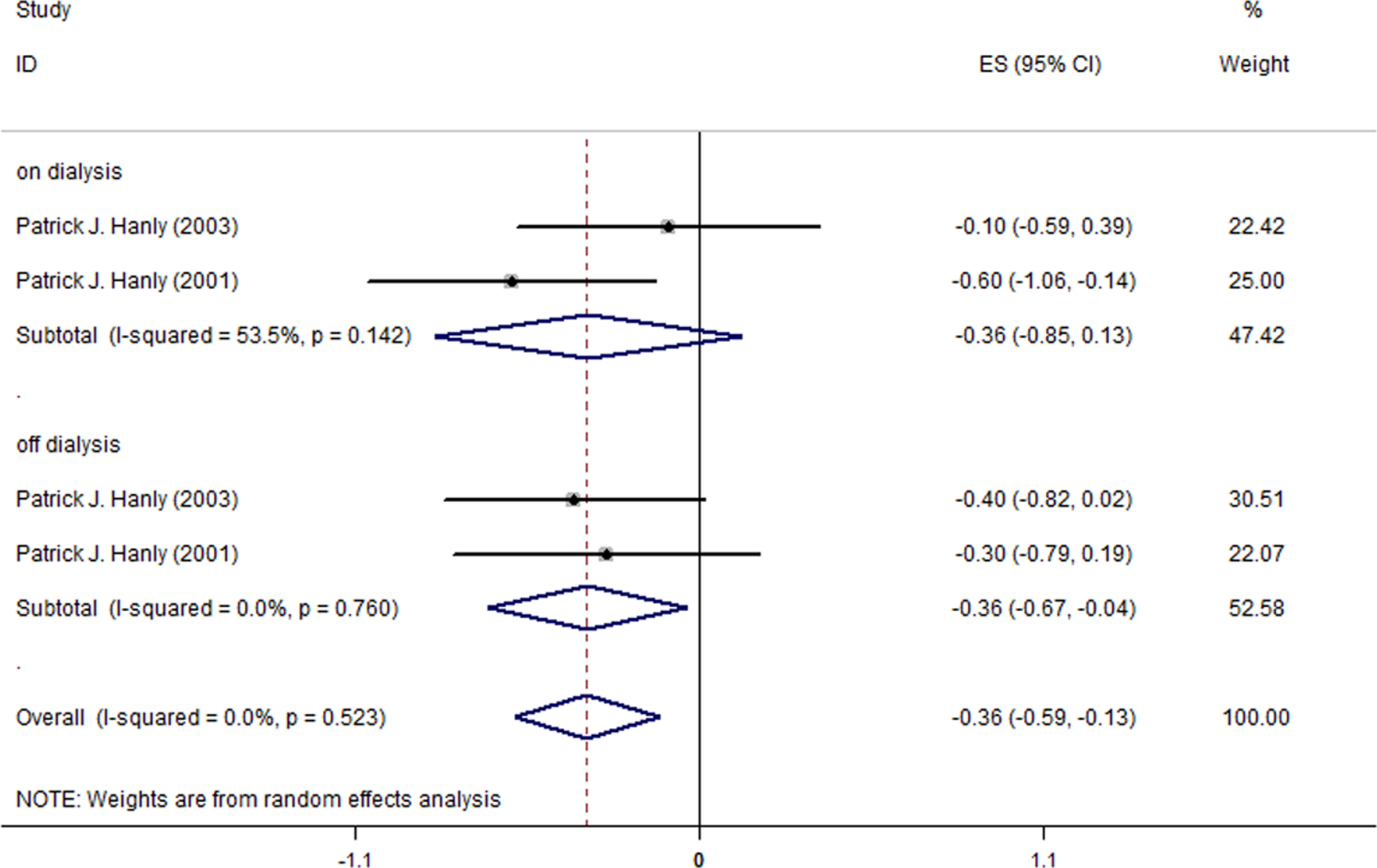
Changes of TST in the 2 included studies for patients with sleep disorders.

For the HD patients with sleep disorders, a similar trend of TST reduction was found after the switch to NHD, and the reduction during off-dialysis days was statistically significant (MD = -0.36, 95% CI, -0.67 to -0.04; I^2^ = 0.0%; Fig 7). However, the reduction during on-dialysis was not statistically significant (MD = -0.36, 95% CI, -0.85 to 0.13; I^2^ = 53.5%; Table 5).

## Discussion

This study has systematically reviewed the effect of NHD on various types of sleep parameters, including AHI, SaO2, TST and sleep-quality-related scores. Some of the sleep parameters (AHI, SaO2, and TST) were measured based on an objective method such as PSG and actigraphy, while some were measured based on subjective sleep questionnaires or interview. We found that sleep assessment by objective methods showed some improvement after a switch to NHD, while the results based on subjective methods still remained inconsistent. Specifically, this meta-analysis found that there was a significant decrease of AHI and increase of SaO2 after a switch from CHD to NHD in HD patients. A negative impact of NHD has been observed through a reduced TST although it is not statistically significant.

AHI has been used as a sleep parameter indicating the severity of sleep apnea in the general population [14]. This meta-analysis shows significantly reduced AHI in hemodialysis patients, and moreover this improvement in AHI is even greater in hemodialysis patients with sleep disorders. It can be explained by several mechanisms. First, Beecroft et al. [8] suggested that the switch to NHD is associated with an increase in pharyngeal cross-sectional area, which may play an important role in the improvement of sleep apnea. It is also found that NHD, compared with CHD, improves ultrafiltration and hemodynamic stability greatly, which is important in reducing extracellular fluid volume [33]. This might eventually correct edema of the pharyngeal wall and para-pharyngeal tissues, which could narrow the airway caused by fluid overload [9, 34]. Second, it is known that ESRD is associated with an increase in respiratory chemoreflex sensitivity to hypercapnia [35] by the metabolic changes which accompany renal failure, such as metabolic acidosis, and certain uremia toxins [36]. The increased responsiveness may promote the destabilization of central respiratory control by facilitating an increased ventilatory response to sufficient ventilatory stimuli [37], and then may contribute to the pathogenesis of sleep apnea in ESRD population [35, 37, 38]. Switching to NHD may decrease chemoreflex responsiveness, and the consequent change in ventilatory sensitivity affects AHI changes [9]. Third, uremic myopathy and uremic neuropathy are common problems in ESRD. Uremic myopathy, which can affect respiratory muscles [39], is known to be associated with accumulation of uremic toxins and malnutrition. Uremic neuropathy involves both sensory and motor neurons that may include the innervation of upper airway dilator muscles [40]. Improved uremic toxins clearance by NHD [41, 42] would increase the strength and endurance of upper airway dilator muscle and correct neuromuscular dysfunction in the upper airway by restoring mechanoreceptor sensitivity to changes in transmural pressure [8]. In turn, the sleep apnea could be reduced.

For SaO2, this systematic review found that a mean SaO2 level among HD patients was below the normal value (i.e., 95%) before switching to NHD. However, after the switch to NHD, SaO2 levels significantly increased to the nominal level. The improvement in SaO2 was greater in patients with sleep disorders. We note that it is unclear whether this association is causal or not due to a significant negative correlation between AHI and SaO2 found in other studies [43]. That is, the decrease of AHI may partially contribute to the increase of SaO2.

The present study suggests that the conversion to NHD might decrease the TST. The performance of a dialysis treatment overnight may disrupt sleep by limiting sleep positions and interrupt the sleep due to alarms; thereby, it could increase anxiety state and decrease sleep quality [44]. Consistent with the decreased TST, frequencies of all arousals (respiratory plus non-respiratory) and periodic leg movements were also reported to remain high [6, 23] although the frequency of respiratory arousals fell significantly after switching to NHD [6]. In other words, NHD might ameliorate signs and symptoms of sleep apnea, but it may not have a net benefit in overall sleep quality [32].

There were several limitations. First of all, due to the paucity of randomized controlled trials, most included studies assessed the effect of NHD with respect to before- and after-switch to NHD within the same patient, lacking an adequate control group. In these types of studies, sleep outcomes among those not switched to NHD cannot be compared. Other drawbacks may include secular trends in treatment and passage of time [45]. However, for its inherent simplicity and accommodation of small sample sizes, this kind of self-control research methodology is commonly used to assess NHD effect in the literature [46]. In addition, previous work has shown that sleep apnea persists on patients consistently using CHD [47]. Second, due to a small number of studies included in our meta-analysis, the statistical power was limited. Even with our best efforts to include more studies, limited studies were available. Third, the high heterogeneity between the pooled effects should not be ignored. We suspect that the dialysis frequency or measurement time might be a potential source of the high heterogeneity. To overcome the limitation, we have conducted additional sensitivity analyses (removing the study that allowed patients to manage their NHD on 3–6 days per week with each session lasting 8 hours [8]) and subgroup analyses (by on- and off-dialysis day). As a result, we have reduced I^2^ index to the levels indicating low heterogeneity.

In conclusion, the study found that conversion from CHD to NHD was associated with an increase of SaO2 as well as a decrease of AHI and TST. For the HD patients, both the decrement in AHI and the increment in SaO2 were larger during on-dialysis days than off-dialysis days. Furthermore, NHD was more beneficial for the patients with sleep disorders. This can be considered as an early evidence that NHD would serve as a better therapeutic alternative for ESRD patients struggling with severe sleep apnea. However, further randomized controlled trials are needed to evaluate the potential benefits of NHD on hemodialysis patients from both subjective and objective perspectives.

## Supporting information

S1 File Search strategy. (DOCX)

S2 File PRISMA checklist. (DOC)
