# Supplementary material for: Effect of nocturnal hemodialysis on sleep parameters in patients with end-stage renal disease: a systematic review and meta-analysis
Source: PLoS One. 2018 Sep 11;13(9):e0203710. doi: 10.1371/journal.pone.0203710 (PMC6133364; doi:10.1371/journal.pone.0203710)
Supplement: S1 File — (DOCX) [file pone.0203710.s001.docx]

**S1 File Search Strategy**

**Search Strategy:**

**Database1: Pubmed**

#1 nocturnal [All fields]

#2 nightly [All fields]

# #3 renal dialysis [MESH Terms]

#4 hemodialysis [All fields]

#5 dialysis [All fields]

#6 #1 or #2

#7 #3 or #4 or #5

#8 #6 and #7

#9 Filters: Publication date to 2018/03/01

**Database 2: Embase <1974 to 2016 Week 19>**

#1 exp nocturnal /

#2 exp nightly /

#3 exp **r**enal **d**ialysis /

#4 exp hemodialysis /

#5 exp dialysis /

#6 #1 or #2

#7 #3 or #4 or #5

#8 #6 and #7

#9 limit #8 to year current
